# Whole Exome Sequencing Study in Isolated South-Eastern Moravia (Czechia) Population Indicates Heterogenous Genetic Background for Parkinsonism Development

**DOI:** 10.3389/fnins.2022.817713

**Published:** 2022-03-17

**Authors:** Kristyna Kolarikova, Radek Vodicka, Radek Vrtel, Julia Stellmachova, Martin Prochazka, Katerina Mensikova, Petr Kanovsky

**Affiliations:** ^1^Department of Medical Genetics, University Hospital Olomouc, Olomouc, Czechia; ^2^Department of Medical Genetics, Faculty of Medicine and Dentistry, Palacký University Olomouc, Olomouc, Czechia; ^3^Department of Neurology, Faculty of Medicine and Dentistry, Palacký University Olomouc, Olomouc, Czechia; ^4^Department of Neurology, University Hospital Olomouc, Olomouc, Czechia

**Keywords:** whole-exome sequencing, parkinsonism, neurodegenerative disorders, trio analysis, *SLC18A2* gene

## Abstract

Parkinsonism belongs to the most common neurodegenerative disease. Genetic predisposition could be one of the significant risk factor for disease development. It has been described higher prevalence of parkinsonism in large pedigree from southeastern Moravia region. The study aims were to select accessible subfamily trios from the pedigree suitable for segregation genetic analyses to perform whole exome sequencing (WES) in trio individuals and further to evaluate genetic variants in the each trio. We used IonTorrent platform for WES for five subfamily trios (1–5). Each trio included two affected and one healthy person (as control). Found variants were filtered with respect to MAF < 1% (minor allele frequency), variants effect (based on prediction tools) and disease filter (Parkinsonism responsible genes). Finally, the variants from each trio were assessed with respect to the presence in the patients. There were found no one founder mutation in the subfamilies from the pedigree. Trio 1 shares two variants with trio 2:*MC1R*:c.322G > A (p.A108T) and *MTCL1*:c.1445C > T (p.A482V), trio 3 shares two variants with trio 5: *DNAJC6*:c.1817A > C (p.H606P) and *HIVEP3*:c.3856C > A (p.R1286W). In trios 4 and 5, there were found two variants in gene *CSMD1*:c.3335A > G (p.E1112G) and c.4071C > G (p.I1357M) respectively. As the most potentially damaging, we evaluated the non-shared variant *SLC18A2*:c.583G > A (p.G195S). The variant could affect dopamine transport in dopaminergic neurons. The study of the parkinsonism genetic background in isolated Moravian population suggested that there could be significant accumulation of many risk genetic factors. For verification of the variants influence, it would be appropriate to perform a more extensive population study and suitable functional analysis.

## Introduction

Parkinson’s disease (PD) is one of the most frequent neurodegenerative disorders. In addition to typical symptoms such as resting tremor, rigidity and bradykinesia, there are other symptoms of parkinsonism: hallucinations, postural instability, dementia etc. (Hughes et al., 2002). The most cases of parkinsonism are sporadic and there is affected about 1,5% population over 65 years. In this case, the disease is probably caused by the combination of genetic, environmental and epigenetic risk factors. The familiar form represents 5–10% cases with Mendelian type of inheritance (Kalinderi et al., 2016). Nowadays, there were described more than 90 genes associated with dominant or recessive inheritance of parkinsonism namely *SNCA* (Campêlo et al., 2017), *LRRK2* (Ortega et al., 2021), *VPS35* (Mir et al., 2018), *Parkin* (Yi et al., 2019; Bandres-Ciga et al., 2020), *DJ-1* (Dolgacheva et al., 2019), *PINK1* (Ando et al., 2017), *DNAJC6* (Köroğlu et al., 2013), *ADH1C* (Buervenich et al., 2005), *PLA2G6* (Lin et al., 2018), *EIF4G1* (Chartier-Harlin et al., 2011), *ATP13A2* (Park et al., 2015) belong to the most important.

The next generation sequencing methods can reveal further genes that could be potential risk factors for parkinsonism.

Despite the many already described causal genes and mutations, genetic predisposition is still unclear in the most of patients. Therefore, it is suitable to change the method strategy from targeted to whole exome (WES) or genome sequencing. It would prepare a field for finding of novel causal or risk genes and variants. *NOTCH4* (Yemni et al., 2019), *TNK2, TNR* (Farlow et al., 2016), *NUS1* (Guo et al., 2018), and *SORL1* (Xiromerisiou et al., 2021) belong to the potential candidate genes recently identified by WES. Combination of WES data and linkage analysis can be used for identification of novel candidate genes in many diseases (Gazal et al., 2016; Toma et al., 2020).

In our previous epidemiology study, we described higher prevalence of parkinsonism in southeastern Moravia region (Hornacko) compared with general population. This region includes 10 villages, where the local people have their own specific traditions (such as dances, folk art, local dialect and religion) and migration out of the region was rare. Due to many years of territorial and social isolation, it was hypothesized that the accumulation of genetic factors may contribute to higher prevalence of PD in the region (Mensikova et al., 2013).

**FIGURE 1:**
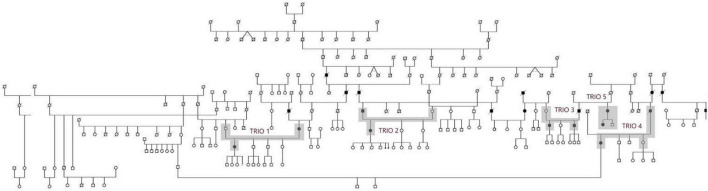
Pedigree of family from Hornacko. Clear circle/square sign living unaffected female/male; black circle/square sign living affected female/male; symbol with a diagonal line is for deceased individual. Highlighted individuals were accessible for WES.

Thanks to our detailed study, 11 generation pedigree from the Hornacko was compiled with the help of witnesses, registry offices and local general practitioners (Figure 1; Mensikova et al., 2013). Based on that, we were looking for patients from pedigree to receive material for genetic analysis. It was possible to select 5 family trios (two affected and one healthy individual) in subfamilies from the large pedigree.

The study aims were to choose accessible trios from the large pedigree suitable for segregation genetic analyses and perform WES and to call and evaluate variants using two software (Ion Reporter and Ingenuity Variant Analysis) and filtering based on genes association with the disease (parkinsonism and other neurodegenerative diseases) and variants co-occurrence in the patients within particular trio and across the whole pedigree.

## Materials and Methods

The study was approved by the Ethics Committee of the Palacký University and University Hospital Olomouc, Czechia. The patients were informed in detail about the study and they all signed informed consent. In our study, 10 patients (8 females and 2 males) and 5 unaffected individuals (3 females and 2 males) were included. The average age of female patients was 67 ± 12.2 years and the average age of male patients was 71 ± 14.1 years. The youngest patient was 56 years and the oldest was 88 years. The average age of controls was 71.8 ± 14.9 years. The youngest control was 51 and the oldest was 87 years. The each trio was composed from 3 family members: two patients and one healthy individual (case assessment is described in Mensikova et al., 2014). We assume autosomal dominant inheritance with reduced penetrance and variable expressivity. The relationships in individual trios: trio 1 (patient number 1 is mother; number 2 is her daughter; control is mother’s brother), trio 2 (patient number 3 is father; number 4 is his daughter; control is father’s brother), trio 3 (patients number 5 and 6 are siblings; control is healthy mother), trio 4 (patient number 7 is mother; number 8 is her daughter; control is mother’s brother), trio 5 (patient number 9 is daughter; number 10 is her mother; control is healthy son). The detailed demographic data are in Table 1. Patients clinical data are described in Table 2. The DNA was isolated in all patients and controls from peripheral blood using salting out method (Miller et al., 1988). WES was performed by commercial company (SEQme, s.r.o., Dobris, Czechia) on Ion Torrent platform. Libraries were prepared using Ion Ampliseq Exome kit (Ion AmpliSeq 2.0 Library, according to manual). Emulsion PCR was done with template kit Ion PI Hi-Q OT2 200. Samples were barcoded to enable to load 3 samples on one Ion PI™ Chip. For sequencing, Ion PI Hi-Q Sequencing 200 kit was used. As reference genome was determinated GRCh37. Sequencing data process includes two parts. For the first part is used Torrent Suite server, where are loaded raw data from sequencer. Raw data (received on the basis of pH change) are converted to single number per well per flow. The next step is base caller, when converted data are translated into base sequence into an unaligned BAM file. For alignment step, there is used Torrent Mapping Alignment Program which performs mapping against reference sequence and it creates BAM files. The second part includes uploading BAM files to Ion Reporter, where is performed variant calling anotation and variants filtering (Vodicka et al., 2020).

**TABLE 1:** Demographic data of patients with neurodegenerative parkinsonism.

	Trio 1	Trio 2	Trio 3	Trio 4	Trio 5
Patients	78 (1, F); 58 (2, F)	81 (3, M); 59 (4, F)	61 (5, M); 56 (6, F)	80 (7, F); 59 (8, F)	58 (9, F); 88 (10, F)
Controls	77 (M)	82 (F)	87 (F)	51 (F)	62 (M)

*Data about age of patients and controls in individual trios, patients’ number in our study and its sex is given in brackets.*

**TABLE 2:** Summary of patient clinical data.

Pat. No.	Gender/age	Age at the disease onset	Clinical phenotype	Clinical signs present at examination
1	F/78	58	PD	Typical rigidity-dominant PD with rigidity, bradykinesia, and rest tremor of upper limbs, advanced stage with the presence of late motor complications (patient treated with DBS)
2	F/58	48	PD	Anosmia lasting about 10 years, clumsiness of the left upper limb, rigidity, and bradykinesia of the left limbs, hypomimia
3	M/81	68	PD	Typical rigidity-dominant PD with rigidity, bradykinesia, and rest tremor of upper limbs, advanced stage with the presence of late motor complications (patient treated with continuous intrajejunal infusion of L-DOPA gel)
4	F/59	53	PD	Hypomimia, clumsiness with rigidity and bradykinesia of the upper limbs predominant to the left
5	M/61	49	PD	Static and resting tremor and rigidity of the upper limbs with a predominance on the right, REM sleep behavior disorder
6	F/56	46	PD	Clumsiness, bradykinesia and rigidity of the right upper limb, postural instability
7	F/died in the 80s	73	DLB	Dementia, mild parkinsonian syndrome
8	F/59	51	PD	Tremor of the right upper limb, rigidity and bradykinesia bilaterally with a predominance of the right, mild cognitive impairment
9	F/58	52	PD	Rigidity and bradykinesia of the right upper limb, hypomimia
10	F/88	70	PDD	Rigidity and bradykinesia bilaterally, asymmetrically with a predominance of the left, postural instability, hypomimia, dysarthria, cognitive deficit

*Pat. No.: number of patient, M: male, F: female, PD: Parkinson’s disease, DLB: dementia with Lewy bodies, PDD: Parkinson’s disease dementia, DBS: Deep brain stimulation, REM: Rapid eye movement.*

The first step of data analysis was selection of variants common only in affected individuals in each trio. All found variants were evaluated and filtered out by two independent software:

1. Ion Reporter - minor allele frequency (MAF) < 1%, Disease research area: Parkinsonian disorders, PD, Neurodegenerative diseases and functional score: SIFT 0.00–0.05 or PolyPhen-2 0.15–1.0 and Grantham 0.0–215.
2. Ingenuity Variant Analysis - minor allele frequency < 1%, variant effects and biological context (Parkinsonism responsible genes).

Genes included in filter Disease research are (Parkinsonian disorders, PD, Neurodegenerative diseases): *ADH1C, AHCY, AP2A2, APBB2, APOD, ARAP2, ATP13A2, ATP6AP2, C9ORF72, CACNA1D, CAPN2, CHCHD2, CHGA, CHMP2B, CKM, CLMN, COQ2, CSMD1, CYP2D6, DCTN1, DNAJC13, DNAJC26, DNAJC6, DNMT1, DRD1, EIF2AK3, EIF4G1, FBXO7, FUS, GALC, GBA, GIGYF2, GNAS, GPC6, GRIA1, HIVEP3, HTR1A, HTRA2, LRRK2, MAPT, MC1R, MOBP, MTCL1, NLRC4, NOS1, PARK10, PARK12, PARK16, PARK3, PARK7, PEPD, PINK1, PLA2G6, PODXL, PRGN, PRKN, PRX, PSD4, PTRHD1, RAB39B, RAB7L1, RIC3, RTN4, SHC2, SLC18A2, SLC20A2, SLC2A3, SLC2A4, SLC6A1, SNCA, SNCB, SORL1, SPTBN2, SQSTM1, STX6, SYNE1, SYNJ1, TARDBP, TBK1, TIA1, TMEM230, TNR, TPPP2, TRIM11, UBB, UCHL1, VCP, VPS13C*, and *VPS35.*

The most important (potentially risk or pathogenic) variants were confirmed by Sanger sequencing.

## Results

In all 15 samples, 99% of targets were covered 1-20× and 90% of targets were covered more than 20×. Average analyzed variants number was about 70,000 with mean depth about 75 (the number of variant in each trio is described in Table 3). The variants potentially associated with neurodegenerative disorders (rare, undescribed, evolutionary conserved and variants assessed by at least one prediction tool as damaging) are described in Table 4. Moreover, we found sharing of some variants within individual trios across the pedigree. In the trio 1 and trio 2 were found two variants in gene *MC1R*:NM_002386.3:c.322G > A (p.A108T) and *MTCL1*:NM_015210.3:c.1445C > T (p.A482V), Trio 3 shares two variants with trio 5: *DNAJC6*:NM_001256864.1:c.1817A > C (p.H606P) and *HIVEP3*:NM_024503.4:c.3856C > A (p.R1286W). In trios 4 and 5, there were found two variants in gene *CSMD1*:NM_033225.5:c.3335A > G (p.E1112G) and c.4071C > G (p.I1357M) rand in the gene *MTCL1*:NM_015210.3:c.1445C > T (p.A482V). In the trio 3 and trio 5 were found two variants in gene *DNAJC6*:NM_001256864.1:c.1817A > C (p.H606P) and in gene *HIVEP3*:NM_024503.4:c.3856C > A (p.R1286W). In the trio 4 and trio 5, there were found two different variants NM_033225.5:c.3335A > G (p.E1112G) and c.4071C > G (p.I1357M) in the same gene *CSMD1.*

**TABLE 3:** Number of variants in individual trios.

Trio	Variants number	Genes number	Number filtered variants and genes	Number variant segregation in affected individuals
Trio 1	72340	16169	25 (21 genes)	12
Trio 2	71471	16372	22 (22 genes)	10
Trio 3	68541	16219	27 (25 genes)	6
Trio 4	67355	16044	25 (25 genes)	4
Trio 5	67589	16143	2 (2 genes)	2

*Variants/genes number is number of all found variants/genes. Number filtered variants/genes is number of found variants/genes after filtering in Ion Reporter software. Number variant segregation is variant number found in affected individuals in each trio.*

**TABLE 4:** Found variants in individual trios.

**Trio 1.**												

**Gene**		**Coordinate**	**Variant**	**Transcript**	**MAF 1000GP/EXAC/gnomAD**	**AA change**	**Rs ID**	**PhyloP**	**LRT**	**SIFT**	**Provean**	**Mutation assessor**

*TNR*		1:175375355	c.496A > G	NM_003285.3	0.0004/0.0044/0.00428	p.T166A	rs147204644	3.788	**9.9999e-7**	0.182	–1.45	1.265
*ADH1C*		4:100268279	c.143G > A	NM_000669.5	0.0020/0.0013/0.0011	p.R48H	rs35385902	2.2479	–	–	–	–0.94
*GRIA1*		5:153026583	c.346G > A	NM_001258022.1	/0.00005/0.0000318	p.V116I	rs138238382	9.6929	**0**	0.426	–0.2	1.78
*TPPP2*		14:21500094	c.371G > A	NM_173846.5	/0.00007/0.0000557	p.S124N	rs755140865	2.6779	**9.9999e-7**	**0.028**	–1.97	**2.565**
*MC1R*		16:89985988	c.322G > A	NM_002386.3	–/–/–	p.A108T	–	–0.536	0.1209	1	0.11	–0.21
*MTCL1*		18:8784555	c.1445C > T	NM_015210.4	–/–/0.000533	p.A482V	rs115077293	0.6178	0.6599	0.219	–1.3	1.04
*APBB2*		chr4:40818179	c.2210A > G	NM_004307.1	0.0052/0.0109/0.013	p.N737S	rs112788816	5.9	**0**	0.953	0.53	–
*SPTBN2*		chr11:66473167	c.1795A > T	NM_006946.2	–/–/–	p.N599T	–	0.05	–	–	–	–
*LRRK2*		chr12:40707778	c.4541G > A	NM_198578.3	0.002/0.0038/0.00526	p.R1514Q	rs35507033	5.841	0.0007759	0.361	–0.68	0.515
*DNMT1*		chr19:10291473	c.206G > A	NM_001130823.2	–/–/0.0101	p.R69H	rs61750053	0.56	0.1205	1	0.04	–

**Trio 2.**												

**Gene**		**Coordinate**	**Variant**	**Transcript**	**MAF 1000GP/EXAC/gnomAD**	**AA change**	**Rs ID**	**PhyloP**	**LRT**	**SIFT**	**Provean**	**Mutation Assessor**

*CAPN2*		chr1:223949314	c.1561G > C	NM_001748.5	0.0058/0.0070/0.00654	p.E521Q	rs28370127	6.216	0.03529	0.491	–1.06	1.475
*RTN4*		chr2:55253903	c.1330_1332delGAT	NM_020532.5	–/0.00004/0.0000439	p.D444del	rs751316044	–0.049	–	–	–	–
*HTR1A;*		chr5:63257465	c.82A > G	NM_000524.3	0.0040/0.0089/0.0094	p.I28V	rs1799921	–0.833	0.4062	0.588	0.11	0
*DRD1*		chr5:174869045	c.1058C > T	NM_000794.5	0.0006/0.0005/0.000461	p.A353V	rs144813919	0.024	0.2128	0.229	–0.88	0.69
*SLC18A2*		chr10:119013618	c.583G > A	NM_003054.4	–/0.000008/0.000012	p.G195S	rs148458078	9.8	**0**	**0.002**	–**5.75**	**2.625**
*GALC*		chr14:88450877	c.443G > C	NM_000153.4	–/0.000008/0.00000401	p.G148A	rs749233234	4.8289	**0.000009999**	0.134	–**4.80**	**2.295**
*MC1R*		chr16:89985988	c.322G > A	NM_002386.3	–/–/–	p.A108T	–	–0.536	0.1209	1	0.11	–0.21
*MTCL1*		chr18:8784555	c.1445C > T	NM_015210.4	–/–/0.000533	p.A482V	rs115077293	0.617	0.6599	0.219	–1.3	1.04
*NLRC4*		chr2:32449832	c.2785G > T	NM_001193513.1	–/–/0.00754	p.A929S	rs61754192	–0.453	0.1116	0.746	–0.03	0.55
*APOD*		chr3:195300740	c.226G > A	NM_001647.3	0.0036/0.0101/0.00953	p.V76M	rs76929107	3.4	**0**	**0.002**	–**2.85**	**3.945**
*ARAP2*		chr4:36230634	c.475C > T	NM_015230.3	–/–/0.00182	p.P159S	rs141442791	1.182	0.3129	0.158	–0.25	1.4
*AP2A2*		chr11:988619	c.1202A > G	NM_001242837.1	0.0012/0.0021/0.00242	p.N401S	rs144441591	8.9	**0**	0.067	–**4.24**	**2.59**
*SLC2A3*		chr12:8074055	c.1445A > G	NM_006931.2	–/0.2705/0.122	p.E482G	rs199523896	6.6	0.2421	0.092	–**2.87**	0
*LRRK2*		chr12:40740686	c.6241A > G	NM_198578.3	0.0098/0.0176/0.0108	p.N2081D	rs33995883	7.1	**0**	0.081	–1.43	0.13
*AHCY*		chr20:32883308	c.112C > T	NM_000687.3	0.0119/0.0042/0.0118	p.R38W	rs13043752	1.6	**0.000088**	**0.001**	–**3.78**	**2.87**

**Trio 3.**												

**Gene**		**Coordinate**	**Variant**	**Transcript**	**MAF 1000GP/EXAC/gnomAD**	**AA change**	**Rs ID**	**PhyloP**	**LRT**	**SIFT**	**Provean**	**Mutation Assessor**

*HIVEP3*		1:42046612	c.3856C > A	NM_024503.4	–/–/–	p.R1286W	rs12132697	1.82	0.1084	**0.004**	–	1.04
*DNAJC6*		1:65858462	c.1817A > C	NM_001256864.2	–/–/0.000277	p.H606P	rs199937139	2.7349	–	0.242	–1.2	–
*PSD4*		2:113940185	c.152G > C	NM_012455.3	–/–/–	p.W51S	–	1.0069	–	**0**	–2.12	0
*CACNA1D*		3:53845162	c.6275G > A	NM_000720.4	–/–/0.0000159	p.G2072E	rs770605004	8.0109	0.03045	**0.012**	–**6.45**	**2.295**
*PEPD*		19:33878972	c.1168G > T	NM_000285.4	–/0.0001/0.00004	p.D390Y	–	5.6719	**0**	**0.008**	–**4.08**	1.58
*CKM*		19:45815121	c.539C > T	NM_001824.5	–/–/0.000124	p.T180M	rs145987658	7.7579	**0.000009**	**0**	–**4.53**	**3.755**
*SLC6A1*		chr3:11070958	c.1243C > A	NM_003042.3	–/–/0.00251	p.L415I	rs112095333	0.521	**0.00007999**	0.245	–0.1	1.095
SYNE1	chr6:152658141	c.12362_12363delAGinsGT	NM_182961.3	–/–/–	p.L4121S	–	–	–	–	–	–
*SORL1*		chr11:121440937	c.3295T > C	NM_003105.5	0.0024/0.0083/–	p.F1099L	rs146903951	6.1669	0.03369	0.477	–0.79	0.055
*CLMN*		chr14:95662946	c.2597A > G	NM_024734.3	–/–/–	pE866G	–	3.77	–	**0**	–**2.69**	–
*PRX*		chr19:40900141	c.4118G > A	NM_181882.2	–/0.00003/0.0000491	p.R1373Q	rs763294661	0.365	0.009036	0.391	–0.34	0.975

**Trio 4.**												

**Gene**		**Coordinate**	**Variant**	**Transcript**	**MAF 1000GP/EXAC/gnomAD**	**AA change**	**Rs ID**	**PhyloP**	**LRT**	**SIFT**	**Provean**	**Mutation Assessor**

*CSMD1*		8:3141748	c.4071C > G	NM_033225.6	–/–/–	p.I1357M	–	0.5329	**0**	**0.002**	–2.01	–
*UBB*		17:16285893	c.672C > T	NM_018955.4	/0.000008/0.0000199	p.R224R	rs771718725	–0.29	–		0	–
*GNAS*		20:57430029	c.1709C > A	NM_080425.3	–/–/–	p.A570D	–	–1.669	0.002453	0.071	–0.39	0.69
*SLC20A2*		chr8:42294592	c.1438G > A	NM_001257181.1	0.006/0.01335/0.0127	p.A480T	rs79577461	0.7279	0.003654	0.508	0.61	–0.51
*SLC2A4*		chr17:7188459	c.1073C > T	NM_001042.2	0.0016/0.0084/0.00597	p.A358V	rs8192702	0.4889	**0.000827**	0.097	–2.37	–
*PLA2G6*		chr22:38528888	c.1027G > A	NM_003560.4	0.0056/0.0185/0.011	p.A343T	rs11570680	1.69	0.004269	0.297	–1.07	0.37

**Trio 5.**												

**Gene**		**Coordinate**	**Variant**	**Transcript**	**MAF 1000GP/EXAC/gnomAD**	**AA change**	**Rs ID**	**PhyloP**	**LRT**	**SIFT**	**Provean**	**Mutation Assessor**

*HIVEP3*		1:42046612	c.3856C > A	NM_024503.4	–/–/–	p.R1286W	rs12132697	1.82	0.1084	**0.004**	–	1.04
*DNAJC6*		1:65858462	c.1817A > C	NM_001256864.2	–/–/0.000277	p.H606P	rs199937139	2.7349	–	0.242	–1.2	–
*EIF4G1*		3:184045397	c.3706C > G	NM_001194947.1	0.0014/0.0032/0.0034	p.P1236A	rs35629949	0.410	–	0.149	–1.27	–
*CSMD1*		8:3205653	c.3335A > G	NM_033225.6	–/–/–	p.E1112G	–	7.8309	**0**	**0.034**	–**5.34**	–
*NOS1*		12:117724018	c.1181A > C	NM_001204218.1	0.0014/0.0043/0.00438	p.D394A	rs9658356	5.076	**9.9999e-7**	0.197	–2.02	0.245
*GPC6*		13:93879805	c.96G > C	NM_005708.5	–/0.0002/0.000204	p.E32D	rs140177257	2.963	**0.000024**	0.318	0.318	1.28
*CHGA*		14:93396098	c.293G > A	NM_001275.4	–/–/0.00557	p.S98N	rs77938104	1.7799	0.004968	**0.024**	–1.35	**2.2**

*Individual variants found in trios 1–5. Coordinate is particular location in genome (related to reference genome GRCh37), MAF 1000GP/ExAC/gnomAD is the minor allele frequency according to 1,000 Genome Project, ExAC (The Exome Aggregation Consortium), gnomAD (The Genome Aggregation Database). AA change is amino acid change, rs ID is the identifier for the described variant, PhyloP score evaluates phylogenetic conservation of particular site in the genome. The colored variants are shared between the individual trios. LRT, SIFT, Provean and Mutation Assesor are prediction tools for variants evaluation. The bold value scores indicate pathogenic variants evaluation. Symbol “–” is used for missing data.*

## Discussion

In our whole exome study, we did not find any founder mutation across the large pedigree. There were analyzed the gene regions known to be associated with parkinsonism.

Further, we found some rare variants which were found in more than one trio and which could contribute to development of neurodegeneration disorders.

***MC1R*** **gene** (OMIM 155555) encodes melanocortin 1 receptor, it is important gene for pigmentation. Study Marti et al. (2015) described *MC1R* gene as a risk factor for PD and association of the variant p.R160W and PD in Spain population (Marti et al., 2015). But this finding was not confirmed (Gan-Or et al., 2016). Individuals with light hair and homozygotes for the variant p.R151C have higher risk for PD compared with wild type (Gao et al., 2009).

The found variant c.322G > A was evaluated as benign according to used prediction tools and the genomic site is phylogenetic unconserved. The missense variant leads to exchange hydrophobic to polar amino acid. The variant has not yet been described.

***MTCL1* gene** (OMIM 615766) encodes protein which is important for microtubule bundles and it interacts with MARK2 (Sato et al., 2013). MARK2 kinase affected affinity of tau protein for microtubules by its phosphorylation (Schwalbe et al., 2013).

According to prediction tools, the found variant c.1445C > T is benign, but its frequency is very rare in population. The genomic site is weakly phylogenetic conserved. There has not been any publication about the variant association with neurodegenerative disorders.

***HIVEP3*** **gene** (OMIM 606649) encodes protein included in HIV enhancer-binding protein family. It can change transcription *via* the κB enhancer motif (Allen et al., 2002). In HIVs patients was described decreased levels of cerebrospinal fluid dopamine (Lopez et al., 1999).

The found variant c.3856C > A was evaluated as benign according to prediction tools LRT and Mutation Assessor, but SIFT evaluated it as damaging. The variant leads to exchange positive charged amino acid to hydrophobic. The genomic site is phylogenetic conserved. Thus, the variant c.3856C > A could affect the protein function.

***DNAJC6*** **gene** (OMIM 608375) is associated with juvenile parkinsonism (Edvardson et al., 2012, Köroğlu et al., 2013). It encodes auxilin which is important for clathrin-mediated endocytosis (Yim et al., 2010). Probably, dysfunction in neuronal endocytic processes could contribute to parkinsonism development.

The found variant c.1817A > C is located in genomic site which is phylogenetic conserved. The variant leads to exchange basic polar amino acid to non-polar. Prediction tools SIFT and Provean evaluated it as benign. The variant frequency is very rare in population.

***CSMD1*** **gene** (OMIM 608397) is associated with schizophrenia risk (Håvik et al., 2011). It is synthetized in developing CNS (central nervous system) and epithelial tissues. The protein is important for complement activation and inflammation in developing CNS (Kraus et al., 2006). Based on WES, *CSMD1* gene was described in association with familial PD (Ruiz-Martínez et al., 2017).

The found undescribed variant c.3335A > G is located in high phylogenetic conserved site and it leads to exchange acid to neutral amino acid. All of used prediction tools evaluated the variant as damaging. Based on that, the variant could leads to affect protein function.

Other found variant c.4071C > G leads to exchange within hydrophobic amino acid in weakly conserved genomic site. The prediction tool Provean evaluated the variant as benign, but LRT and SIFT evaluated it as damaging.

***SLC18A2* gene** (OMIM 193001, also known as *VMAT2*) encodes ATPase antiporter transmitting monoamines- serotonin, dopamine and norepinephrine into vesicles to transport them out of cell (Liu and Edwards, 1997). Increased cytosolic dopamine and its metabolites are neurotoxic for neuronal cells (reduction leads to neuroprotection) (Mosharov et al., 2009). Brighina et al. (2013) described two SNPs in the *VMAT2* promoter region in connection with a reduced risk of PD. It is assumed that increased levels of VMAT2 contribute to protection against the disease (Brighina et al., 2013).

The variant c.583G > A is located in high phylogenetic conserved genomic site and it is very rare in population. It leads to exchange hydrophobic to polar amino acid. All of used prediction tools evaluated it as damaging. In project GnomAD exomes, there were no found homozygous allele, it could indicates likely pathogenic effect (according to ACMG Classification, criteria for classifying pathogenic variants – PMS2 rule). The variant is located in disulfide bond domain, which is important for efficient monoamine transport (Thiriot et al., 2002). We assume that the variant could affect dopamine transport from dopaminergic cells and expose the cells to dopamine cytotoxicity.

## Conclusion

The WES could contribute to the finding of variants responsible for development of many diseases. Generally, the evaluation of variants from the WES using different prediction tools is often not uniform and the final assessment should be taken with caution and in combination with functional assays or segregation analysis. However, even one prediction tool, strong evolution conservation or population rarity could indicate and highlight potentially risk variant.

Based on the study result, we suppose heterogenous genetic background in the development of parkinsonism in Hornacko region. According to the prediction tools, the most interesting variant seems to be *SLC18A2*:NM_003054.4 c.583G > A (p.G195S, rs148458078).

Our study is limited by amount of samples and it is not possible to exclude the effect of other genetic causes that were not detected by used method and filter setting. The WES cannot capture intronic variants and large genomic rearrangements. The larger population study is necessary for verification of our results.

## Data Availability Statement

The datasets presented in this study can be found in online repositories. The names of the repository/repositories and accession number(s) can be found below: https://www.ncbi.nlm.nih.gov/bioproject/785725.

## Ethics Statement

The studies involving human participants were reviewed and approved by the Palacký University and University Hospital Olomouc. The patients/participants provided their written informed consent to participate in this study.

## Author Contributions

KK and RVr: trio analysis and evaluation of variants. RVo: trio analysis and evaluation of variants and coordination of writing manuscript. JS: genetic consultation of patients. MP: genetic consultation of patients and coordination of writing manuscript. KM: neurological consultation of patients. PK: neurological consultation of patients and coordination of writing manuscript. All authors contributed to the article and approved the submitted version.

## Conflict of Interest

The authors declare that the research was conducted in the absence of any commercial or financial relationships that could be construed as a potential conflict of interest.

## Publisher’s Note

All claims expressed in this article are solely those of the authors and do not necessarily represent those of their affiliated organizations, or those of the publisher, the editors and the reviewers. Any product that may be evaluated in this article, or claim that may be made by its manufacturer, is not guaranteed or endorsed by the publisher.
